# AN INNOVATIVE APPROACH TO EXPANDING AGING SERVICES ACCESS IN RURAL COMMUNITIES: RURAL AGING ACTION NETWORK

**DOI:** 10.1093/geroni/igae098.2519

**Published:** 2024-12-31

**Authors:** Molly Wylie, Ashley Washington, Verena Cimarolli, Robyn Stone

**Affiliations:** LeadingAge LTSS Center @UMass Boston, Santa Monica, California, United States; Lutheran Services in America, Washington, District of Columbia, United States; LeadingAge, Washington, District of Columbia, United States; LeadingAge, Washington, District of Columbia, United States

## Abstract

A significant share of older adults live in rural areas and wish to remain in their homes and communities. Aging in rural communities poses unique challenges that can limit access to healthcare, transportation, and other services that promote independence. To address these challenges, Lutheran Services in America (LSA) developed Rural Aging Action Network (RAAN), an innovative collaborative that leverages existing community-based services and supports for older adults who are underserved in rural communities of Minnesota, Montana, North Dakota, and South Dakota. A mixed-method evaluation of RAAN was conducted over a three-year period to assess the extent of community need, the network’s effectiveness in addressing care gaps, and lessons learned in implementing RAAN. Descriptive analyses of client-level data showed that 483 older adults have been in contact with the network, and over half (55%) were referred to services. Chi-square tests indicated that older adults with restricted incomes were significantly more likely to be referred to any service than older adults whose incomes meet their needs (69% versus 31% respectively, X2 (1, N = 258) = 9.8, p =.002)), suggesting RAAN’s effectiveness in reaching low-income populations. Focus groups and interviews with 12 older adults and 9 caregivers found that transportation, technology training, and English translation remain service gaps in the community. Respondents expressed the value of RAAN in raising awareness about available services and providing a one-stop-shop for aging resources. This evaluation can help inform the implementation of similar networks in rural communities nationwide.

